# Removal of Cherry-Stones from the Rectum

**Published:** 1876-03

**Authors:** William H. Westcott

**Affiliations:** Dorchester


REMOVAL OF A GREAT NUMBER OF CHERRY-STONES FROM THE RECTUM—*By William H. Westcott, M. D., of Dorchester.*—In July, 1875, I was called to see a boy about eight years old who had complained for several days of tenesmus, but had passed nothing, except occasionally a small amount of mucus tinged with blood. He had taken several doses of cathartic medicine, with no result other than severe griping and increased tenesmus. The mother stated that she could not pass the nozzle of a syringe more than an inch into the rectum. I found the bladder enormously distended, and attempted at once to introduce the catheter, but its course was suddenly arrested.

Upon passing a finger into the rectum I found it distended with an unyielding mass of cherry-stones, filling the whole pelvic cavity and pressing the urethra against the pubic arch. The cherry-stones were so firmly glued together that it was quite difficult to separate them by the finger. After about one hundred of the stones had been removed, one by one, by means of ordinary dressing forceps, the boy passed a large quantity of urine. Palpation discovered numerous masses of the stones in the large intestines.

The next morning the rectum was packed quite full again, and the boy could not pass his water. I administered ether, and removed all the stones within reach. The boy soon urinated, and about an hour later had a movement of the bowels, passing many more stones, which were not preserved. After a few days the boy was quite well.

The case was reported some time ago to the Boston Society for Medical Improvement, and the stones, which were then exhibited (measuring six ounces and six drachms), have been sent to the museum of the medical college.—*Boston Medical and Surgical Journal.*

